# Metastatic Breast Carcinoma to the Prostate Gland

**DOI:** 10.1155/2016/8264140

**Published:** 2016-06-26

**Authors:** Meghan E. Kapp, Giovanna A. Giannico, Mohamed Mokhtar Desouki

**Affiliations:** Department of Pathology, Microbiology and Immunology, Vanderbilt University Medical Center, Nashville, TN 37232, USA

## Abstract

Cancer of the male breast is an uncommon event with metastases to the breast occurring even less frequently. Prostate carcinoma has been reported as the most frequent primary to metastasize to the breast; however, the reverse has not been previously reported. Herein, we present, for the first time, a case of breast carcinoma metastasizing to the prostate gland. Prostate needle core biopsy revealed infiltrative nests of neoplastic epithelioid cells, demonstrated by immunohistochemistry (IHC) to be positive for GATA3 and ER and negative for PSA and P501S. A prostate cocktail by IHC study demonstrated lack of basal cells (p63 and CK903) and no expression of P501S. The patient's previous breast needle core biopsy showed strong ER positivity and negative staining for PR and HER2. Similar to the prostate, the breast was negative for CK5/6, p63, and p40. This case demonstrates the importance of considering a broad differential diagnosis and comparing histology and IHC to prior known malignancies in the setting of atypical presentation or rare tumors.

## 1. Introduction

Cancer of the male breast represents less than 1% of all breast cancers in the United States and incidence is increasing with recent approximations of 1.3/100,000 [1, 2]. While most men who develop breast cancer have no recognized risk factors, a minor subset have testicular damage (mumps, undescended testes, and high ambient working temperature). Risk has also been associated with increased body mass index, gynecomastia, increased serum estradiol level, and diabetes [3, 4]. Though formal screening programs are not established for men, most present with early stage I or II. Stage at diagnosis is a strong prognostic factor and men with triple-negative breast cancer have a worse prognosis [5].

Interestingly, men with ER-positive cancer are reported to have a 30% reduction in risk of death compared with ER-negative breast cancer; however, that benefit applies only to the first 5 years from diagnosis, at which time ER positive and negative have a similar prognosis [5]. Therapy for male breast cancer has mostly been extrapolated from treatment trials for female breast cancer, which has been shown to be ineffective. Important differences have emerged, including that male breast cancer prognosis is significantly better after adjuvant treatment with tamoxifen compared to an aromatase inhibitor, and that male breast cancer is not congruent with female breast cancer [6].

While primary carcinoma of the male breast is infrequent, metastatic carcinoma to the breast from distant organs is also very rare comprising approximately 1.2–2.7% of all malignant breast tumors [7] with the prostate being the most common primary site [8]. While breast is an exceptional site of prostatic carcinoma metastasis, it is a documented phenomenon, whereas the reverse has not been described. Herein, we present a case of metastatic breast carcinoma to the prostate of a 63-year-old male. To the best of our knowledge, this is the first-case report of breast carcinoma metastatic to the prostate.

## 2. Case Report

### 2.1. Clinical Presentation

The patient is a 63-year-old male who presented with a newly diagnosed neoplasm of the prostate. His past medical history was significant for Bowen's disease status after excision, and breast cancer status postmastectomy and axillary dissection. The patient began to experience increasing lower urinary tract symptoms, manifested as urinary hesitancy, weak force of stream, and subjective sensation of incomplete bladder emptying. A rectal examination revealed a firm prostatic nodule in the context of a PSA of 0.88.

### 2.2. Radiological Findings

CT scan with contrast of the chest demonstrated mediastinal and bilateral hilar lymphadenopathy, the dominant lymph node measuring 1.8 cm in greatest dimension. Multiple bilateral pulmonary masses, some of which appeared spiculated were identified. The dominant spiculated mass of the left lower lobe measured 2.4 cm in greatest dimension. The CT of the abdomen and pelvis identified two metastatic nodules in the omentum (1.6 cm in greatest dimension). The prostate was notably enlarged, heterogeneously enhancing, and bulging into the bladder base, which demonstrated mild thickening of its wall.

### 2.3. Surgery

Following needle core biopsies of his prostate, and due to high tumor density reported within the prostate, the patient was scheduled for a transurethral resection of prostate (TURP). and started on tamoxifen hormone therapy. Urinary retention was managed with self-catheterization; however, he experienced frequent inability to fully empty his bladder due to clots. Gross hematuria developed and TURP procedure was performed.

### 2.4. Pathology

#### 2.4.1. Breast

**Figure 1:**
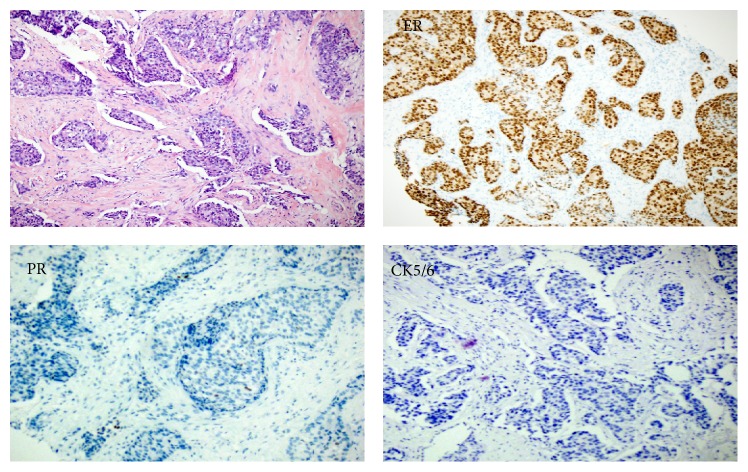
Breast core biopsy shows infiltrative nests of epithelioid cells with small ovoid hyperchromatic nuclei and modest eosinophilic cytoplasm separated by fibrous stroma with a desmoplastic reaction on H&E. Immunohistochemical stains show nuclear staining for ER and negative staining for PR and CK5/6.

Ultrasound guided needle core biopsy and simple mastectomy showed invasive mammary carcinoma, no special type, with high combined histologic grade and intermediate proliferative rate. The mass was 2.6 cm in greatest extent and margins on the mastectomy were negative for malignancy. Submitted immunohistochemistry (IHC) stains (Figure 1) showed the tumor cells to be ER positive (strong, 98% of neoplastic nuclei) and negative for progesterone and HER2.

#### 2.4.2. Prostate Biopsies

**Figure 2:**
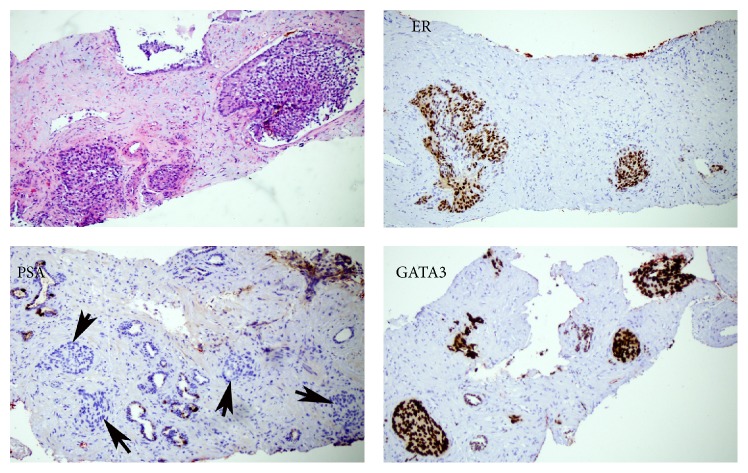
Prostate needle core biopsy shows infiltrative nests of hyperchromatic nuclei with modest eosinophilic cytoplasm separated by fibrous stroma with a desmoplastic reaction on H&E. Immunohistochemical stains show nuclear staining for ER and GATA3 and negative staining by PSA.

Microscopic examination of the needle core biopsies from the prostate gland demonstrated a high burden of infiltrating neoplastic cells. IHC studies (Figure 2) showed the tumor cells to be positive for GATA-3 and to have strong positive ER nuclear staining in 100% of neoplastic nuclei. PSA and P501S were negative. A prostate immunohistochemical cocktail demonstrated a lack of basal cells (P63 and CK903) and no expression of P504S. In the setting of the patient's history and the characteristic morphology and immunoprofile, metastatic breast cancer was favored; however, urothelial carcinoma could not be entirely excluded [9]. IHC stains performed on both the breast and prostate biopsies showed a similar pattern of staining: negative CK5/6, p63, and p40.

#### 2.4.3. Prostate Transurethral Resection

Grossly, the specimen consisted of multiple soft, pink-tan fragments of tissue admixed with blood clot weighing 9.2 grams and measuring 3.9 × 3.4 × 0.9 cm in aggregate. The specimen was entirely submitted for histopathologic review. Light microscopic evaluation demonstrated metastatic carcinoma, morphologically consistent with breast primary, similar to previous prostate biopsies and breast excision.

### 2.5. Management and Follow-Up

The patient received tamoxifen therapy, but his disease progressed rapidly. Brain metastases evidenced by 1.1 cm homogeneously T2 hyperintense rounded focus at the gray-white junction within the left frontal lobe and a second more ill-defined focus in the right paramidline parietal lobe posteriorly clinically manifested with headache. Ophthalmologic evaluation of the patient's endorsement of blurry vision in the right eye, in addition to multiple new floaters and persistent flashing lights revealed creamy-white subretinal placoid lesions in association with serous exudative retinal detachment inferiorly concerning for metastatic disease. The patient's last hospitalization for diabetic ketoacidosis, septicemia, and urinary tract infection secondary to* E. faecalis* and fungemia was complicated by cardiopulmonary arrest secondary to thromboemboli, confirmed by postmortem examination.

## 3. Discussion

The involvement of the prostate gland by metastasis from noncontiguous tumors is a rare occurrence reported in <1% of surgical specimens and 3% of postmortem examinations [10, 11]. Secondary tumors of the prostate have been noted with most frequency from the digestive tract [12, 13], lung [14, 15], and kidney [16, 17]. Up to 20% of patients with secondary tumors of the prostate have no evidence of metastatic disease in additional sites [11].

Urothelial carcinoma may be a secondary tumor of the prostate and is a relatively common finding in advanced-stage bladder disease [10]. GATA-binding protein 3 (GATA3) is a highly sensitive and reproducible biomarker of urothelial differentiation; however, breast epithelial cells have also been shown to stain with GATA-3, rendering this IHC stain incapable of deciphering the two differentials in our case [18]. As morphology and initial IHC evaluation (GATA-3 and ER positivity) could not completely exclude urothelial primary with prostatic duct involvement, morphologic, and IHC comparison of the patient's primary breast cancer and current prostatic neoplasm was necessary [9, 18]. The comparison showed similar morphology and pattern of staining, thereby concluding prostatic involvement by metastatic breast carcinoma.

In the reported case, the correct diagnosis of metastatic breast cancer was important given the significant difference in management between patients with primary prostatic carcinoma, metastatic urothelial carcinoma, and breast cancer. For example, hormone therapy (tamoxifen) can be utilized as the first-round therapy for metastatic breast carcinoma with ER positivity [6]. In conclusion, this is the first reported case of metastatic breast cancer resulting in high disease burden of the prostate, and it demonstrates the importance of a broad differential diagnosis, patient history, and access to previous material for comparison in the setting of rare tumor or atypical presentation.

## Abbreviations

IHC: Immunohistochemistry
ER: Estrogen receptor
PR: Progesterone receptor
TURP: Transurethral resection of the prostate.
